# Practical Applications of Self‐Healing Polymers Beyond Mechanical and Electrical Recovery

**DOI:** 10.1002/advs.202302463

**Published:** 2024-02-15

**Authors:** Semin Kim, Hyeonyeol Jeon, Jun Mo Koo, Dongyeop X. Oh, Jeyoung Park

**Affiliations:** ^1^ Research Center for Bio‐based Chemistry Korea Research Institute of Chemical Technology (KRICT) Ulsan 44429 Republic of Korea; ^2^ Department of Organic Materials Engineering Chungnam National University Daejeon 34134 Republic of Korea; ^3^ Department of Polymer Science and Engineering and Program in Environmental and Polymer Engineering Inha University Incheon 22212 Republic of Korea; ^4^ Department of Chemical and Biomolecular Engineering Sogang University Seoul 04107 Republic of Korea

**Keywords:** artificial intelligence, commercialization challenges, innovative applications, self‐healing materials, self‐healing polymers

## Abstract

Self‐healing polymeric materials, which can repair physical damage, offer promising prospects for protective applications across various industries. Although prolonged durability and resource conservation are key advantages, focusing solely on mechanical recovery may limit the market potential of these materials. The unique physical properties of self‐healing polymers, such as interfacial reduction, seamless connection lines, temperature/pressure responses, and phase transitions, enable a multitude of innovative applications. In this perspective, the diverse applications of self‐healing polymers beyond their traditional mechanical strength are emphasized and their potential in various sectors such as food packaging, damage‐reporting, radiation shielding, acoustic conservation, biomedical monitoring, and tissue regeneration is explored. With regards to the commercialization challenges, including scalability, robustness, and performance degradation under extreme conditions, strategies to overcome these limitations and promote successful industrialization are discussed. Furthermore, the potential impacts of self‐healing materials on future research directions, encompassing environmental sustainability, advanced computational techniques, integration with emerging technologies, and tailoring materials for specific applications are examined. This perspective aims to inspire interdisciplinary approaches and foster the adoption of self‐healing materials in various real‐life settings, ultimately contributing to the development of next‐generation materials.

## Graphical abstract

Most studies primarily focus on the mechanical protection of objects based on the self‐recovery of wounds. However, this is the tip of the iceberg for self‐healing materials. Several researchers are beginning to explore and develop novel applications. In this perspective, an overview of these innovative applications is provided and directions for future research on self‐healing materials are proposed.

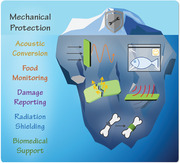

## 1 Introduction

Self‐healing polymers have emerged as a groundbreaking area of research in materials science and engineering, owing to their ability to repair damage thereby extending the life cycle of products and reducing plastic waste and costs.^[^ ^1^ ^]^ Consequently, self‐healing polymers have attracted considerable attention from both academia and industry. Researchers have investigated the use of self‐healing polymeric materials in various applications.^[^ ^2^, ^3^, ^4^, ^5^, ^6^, ^7^, ^8^, ^9^, ^10^ ^]^ Scientific approaches include the development of innovative chemistries, such as reversible covalent bonds and supramolecular chemistry and their incorporation into diverse polymer structures such as hydrogels, elastomers, vitrimers, and even composites. Engineering approaches have focused on the optimization of protective materials such as concrete for construction, paint protection films for vehicles, and housing for advanced devices, including wearable devices and soft robotics.

Numerous comprehensive studies have been conducted on contemporary self‐healing polymers and their applications, primarily focusing on their mechanical properties and durability from a materials science perspective and housings and coatings for electronics from an application standpoint.^[^ ^11^, ^12^, ^13^, ^14^, ^15^, ^16^, ^17^ ^]^ However, from an industrial perspective, this field remains relatively unexplored. Despite the substantial contributions of self‐healing polymers in academia, their industrialization and commercialization remain largely unrealized.^[^ ^18^ ^]^ For instance, Nissan Motor and Bayer Corporates have announced the commercialization of self‐healing clear coat paint to protect vehicle surfaces according to their claim,^[^ ^19^, ^20^ ^]^ while the exact scientific technology has not been clearly revealed. The wider adoption of advanced intrinsic self‐healing polymers still faces challenges such as difficulties in scaling up complex chemistries, lower robustness than that of conventional materials, and performance degradation at extreme temperatures. To overcome these limitations, self‐healing materials must be sufficiently robust for broader applications. In the outlook section, research trends for enhancing material performance, such as incorporating nanomaterials and stimuli‐responsive systems, are introduced.

Furthermore, to facilitate the commercialization of self‐healing materials, their applications should extend beyond coatings and housings to advanced materials for use in various fields including energy storage, aerospace, and biotechnology, thereby accelerating commercialization through market expansion. Previous review articles have primarily addressed product longevity and mechanical stability or electrical performance resulting from the use of self‐healing materials. However, this is a rather narrow perspective on the potential of these materials. Indeed, more sophisticated applications can be realized by considering the physicochemical characteristics of self‐healing. First, from a thermodynamic standpoint, self‐healing is a phenomenon that reduces the interfacial area, indicating that self‐healing materials can potentially act as sensors or actuators that respond to changes in temperature, humidity, and pressure. Second, because self‐healing involves rheological energy storage, self‐healing materials can be utilized for physical energy conversion, such as storing elastic energy from mechanical movements. Third, self‐healing is typically associated with living organisms rather than inanimate objects, suggesting that self‐healing materials can play a complementary role in regenerating physical damage, potentially revolutionizing medical implants and tissue engineering. Finally, during product manufacturing, self‐healing materials can be seamlessly attached without the need for adhesives, resulting in improved 3D printing processes and the elimination of interfaces between printed layers, ultimately achieving smooth, continuous production.

**Figure 1:**
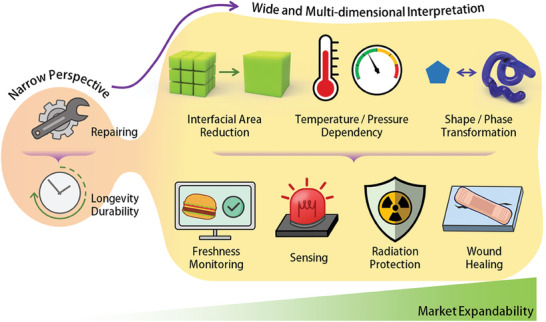
Market expandability by application diversification based on multidimensional interpretation of self‐healing behaviors.

In this perspective article, our objective is to provide a comprehensive summary of research articles that investigate practical high‐level applications of self‐healing materials, particularly those that demonstrate advanced real‐life applications achieved through a multidimensional interpretation of the self‐healing phenomenon (**Figure** **1**). Some of these studies have explored research themes that utilize self‐healing behavior as a critical mechanism driving various applications including food packaging with antimicrobial properties, damage‐reporting for structural safety, radiation shielding for space exploration, acoustic conservation for noise reduction, biomedical monitoring with intelligent drug delivery systems, and tissue regeneration for wound healing.

The authors of these articles found appropriate applications that fit the different properties of the materials they developed. As discussed above, we introduce material research strategies for the successful commercialization and industrialization of self‐healing materials, delving into several key areas such as novel healing mechanisms, enhanced healing efficiency, scalability, and affordability. Furthermore, we examine these developments and the potential future impact of self‐healing materials. The most promising research directions include promoting environmental sustainability, harnessing advanced computational techniques, integrating with emerging technologies, and tailoring materials for specific applications. By emphasizing the practical applications of self‐healing materials in these areas and exploring innovative interdisciplinary approaches, we hope to foster their adoption in real‐life settings, deliver significant benefits, and contribute to the development of next‐generation materials.

## 2 Broader View of the Self‐Healing Phenomenon, and the Expansion of Its Potential Applications

**Figure 2:**
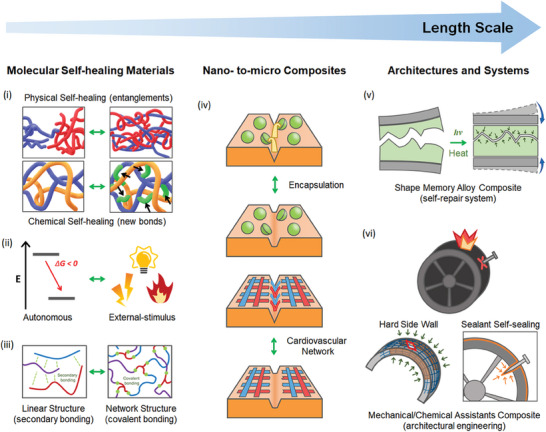
Six categorization stems of self‐healing behavior by length scale: i) physical and chemical healing, ii) dependency on stimuli, iii) types of molecular structure in molecular self‐healing materials, iv) microcapsule and cardiovascular network containing healing agents in nano‐to‐micro scale composites, v) physical contact/diffusion support, and vi) collapse‐resistant engineering in architectural and systematic self‐healing (self‐repair) with supporting materials.

Self‐healing materials have gained considerable interest due to their ability to repair damage, thereby enhancing the lifespan and performance of various products. These materials can be broadly categorized into six types, according to three distinct criteria: the scale of self‐healing drivers (molecular systems, nano‐to‐micro composites, and millimeter‐to‐centimeter architectures), dependency on stimuli (autonomous in ambient conditions vs stimulus‐dependent), and molecular structure (linear vs networked polymers). This discussion is organized based on the scale of self‐healing drivers, the largest categorization stem (**Figure** **2**).

Starting with the smallest scale, molecular self‐healing materials inherently repair damage through the use of dynamic chemical bonds within their structures. These types of self‐healing materials utilize various types of dynamic covalent bonds (e.g., Diels–Alder reactions, radical recombination, urea chemistry, olefin metathesis, polysiloxanes, boronic esters, acylhydrazones, and other reactions) and/or supramolecular interactions (e.g., hydrogen bonds, metal–ligand interactions, and host–guest interactions).^[^ ^21^, ^22^, ^23^, ^24^, ^25^, ^26^, ^27^, ^28^, ^29^, ^30^ ^]^ In these materials, fractures are repaired when dynamic bonds at the interface form new connections, effectively repairing the damage. Thermodynamically, new bonds are formed by reversible bonding at the damaged interface, and kinetically, the formation of new bonds at the interface is proportional to the diffusion rate of polymer chains. Therefore, they are often used in elastomeric materials, which are polymeric systems with relatively high diffusion rates, providing potential applications in protective coatings or laminates. The advantage is their ability to operate repeatedly without exhausting external additives.

Molecular self‐healing materials are commonly divided into two types based on their dependency on external stimuli. The former type is autonomous self‐healing materials, which operate under ambient conditions. In these materials, dynamic bonds function efficiently at temperatures close to room temperature, supported by a sufficient diffusion rate of the polymer chains. However, the downside of having such a high diffusion rate at room temperature is that the material can become limp. Although autonomous materials have the advantage of self‐sufficiency, they often face a trade‐off between the self‐healing rate and mechanical strength. The most ideal form of self‐healing material would have the mechanical strength issue resolved. The latter type consists of materials that require external stimuli, such as light, heat, or moisture, for self‐healing. From a thermodynamic perspective, the dynamic bonds within these materials are activated or catalyzed by an external trigger. Alternatively, from a kinetic standpoint, an external energy input may be necessary to facilitate chain diffusion, particularly when the material is inherently rigid. Specific reactions, such as aliphatic disulfide or Diels–Alder reactions, require heat to induce chemical bond exchange. Despite the apparent disadvantage of requiring external intervention for self‐healing, these materials offer the benefit of controlled healing/irradiation, acting as switches to initiate self‐healing when required.

Molecular self‐healing materials are also subdivided into two categories based on their molecular topology. Conventional self‐healing polymers typically feature a linear chain structure. There are exceptions in the form of cross‐linked self‐healing rubbers or elastomers; however, their cross‐linking density is low enough not to considerably increase the Young's modulus of the elastomers. A unique class of materials in this context is covalent adaptable networks (CANs).^[^ ^31^, ^32^, ^33^, ^34^, ^35^, ^36^, ^37^, ^38^ ^]^ These materials display a distinctive property: above a specific temperature, they become malleable and can be reshaped, repaired, or even recycled at above a certain temperature. This unusual behavior stems from the dynamic covalent networks within the CANs, which allow the polymers to alter their topology. As a result, CANs exhibit a balance between 1) the robustness and rigidity typical of thermoset polymers; and 2) the added benefit of reprocessability.

CANs could be classified into two groups depending on the exchange mechanism: 1) associative networks (referred to as “vitrimer”)^[^ ^39^, ^40^, ^41^, ^42^, ^43^, ^44^, ^45^ ^]^ and 2) dissociative networks.^[^ ^46^, ^47^, ^48^, ^49^ ^]^ Both are akin to thermoset polymers. Specifically, vitrimers are distinguished from dissociative CANs and thermoplastic polymers. The viscosity of vitrimers resembles typical inorganic silica materials. It appears that the relationship between viscosity and temperature follows the Arrhenius law as temperature increases. This is because the crosslinking density is maintained, unlike dissociative CANs that lose network integrity. In dissociative CANs, the self‐healing behavior occurs with the breaking of chemical bonds first, followed by the formation of new bonding. However, in vitrimers, chemical bonding is broken simultaneously as new covalent bonding is formed. For the dissociative CANs, the equilibrium state between dissociated and associated crosslinking density is the function of temperature. It means that the crosslinking density could be controlled by temperature adjusting, depending on whether it is exothermic or endothermic reaction.^[^ ^50^ ^]^

Stepping up the scale, self‐healing nano‐to‐micro composites employ encapsulated healing agents within the material's structure.^[^ ^51^, ^52^, ^53^, ^54^, ^55^ ^]^ These healing agents are often housed in structures such as microcapsules or vascular networks, ranging in size from the nano to micrometer scale. When damage occurs, these liquid‐like healing agents are released into the affected area, filling the cracks and fractures with substances like adhesives, curing agents, or other suitable materials. One advantage of these types of self‐healing materials is that they are less reliant on the thermodynamic or kinetic properties of the substrate polymeric material, which means that they do not require a low Young's modulus for efficient healing. As a result, they are particularly suited to harder, glassy materials including thermosetting resins and cement materials—areas where molecular self‐healing materials often fall short. However, self‐healing composite systems also face challenges. These include issues with scalability, the long‐term retention of healing agents, and durability, all of which can affect their effectiveness in practical applications. These problems often arise from the leakage and dispersion of healing agents over extended periods.

Last, there are self‐healing materials that are engineered on a macro‐scale, from millimeters to centimeters. These materials have been designed and produced using principles from mechanical or architectural engineering.^[^ ^56^, ^57^, ^58^ ^]^ A well‐documented material is a composite with shape‐memory alloy (SMA) wires inserted between layers of plastic resin. In the event of a crack, applying heat causes the SMA wires to return to their original state, effectively closing the crack. Another example originates from the tire industry, where companies such as Hankook Tire Corporate (South Korea) have developed self‐sealing tires. The surface of the tires is constructed by layering rubber materials with varying viscoelastic properties. The comparatively harder rubber at the surface undergoes the event of a puncture by a nail, while the more ductile and tough rubber underneath effectively seals the hole, preventing further puncturing or damage. Notably, while the molecular‐, nano‐, and micro‐scale materials discussed above still require a great deal of research before they can be fully commercialized, these macro‐composites use the existing commercialized materials from a design perspective. Materials designed with this larger macro‐structure are currently the most successful demonstration due to their utilization of a combination of commercially available materials.

Although these six different self‐healing materials are often used as protective coatings or laminates, their potential applications extend far beyond the thermodynamic and rheological aspects of self‐healing. Particularly, molecular self‐healing materials present a broader application spectrum due to their responsiveness to external stimuli. This characteristic will be explored in more detail as we delve into the emerging applications of these molecular self‐healing materials

First, the self‐healing process is influenced by factors such as temperature, humidity, and pressure, which affect the healing rate and phase transition together with crystallinity, interfacial area, and volume changes. Owing to this sensitivity, self‐healing materials can potentially function as sensors or actuators, detecting external factors by monitoring changes in the specific surface area in response to these variables.

Second, self‐healing materials can be employed for physical energy conversion because this process involves rheological energy storage and loss. This unique property allows these materials to dissipate mechanical stress or restore potential elastic energy. This ability is driven by mechano‐responsive phase changes and alterations in the molecular structure for self‐healing. From a rheological standpoint, self‐healing process is initiated when a material's liquid properties eclipse its solid attributes, typically observed under conditions of a low relaxation time, signifying an elevated potential for self‐healing. Nevertheless, it is essential to recognize the dichotomy between self‐healing ability and mechanical resilience. An amplification in liquid properties correlates with a diminution in mechanical strength. To surmount this obstacle, researchers have pioneered mechano‐responsive self‐healing systems. Within these systems, the dynamic bonds responsible for the repair mechanism exhibit increased liquid‐like characteristics when in a relaxed state due to the effective exchange between bonding and de‐bonding states. However, under stress, the polymer chains of mechano‐responsive materials undergo phase transitions, such as phase separation,^[^ ^59^ ^]^ meta‐stable crystal formation,^[^ ^60^ ^]^ and ionic complexation.^[^ ^61^ ^]^ These phase transitions are fueled by the absorption of external mechanical energy. Upon the removal of the external stress, the material reciprocates by restoring its original strain, shape, and molecular structure, thereby releasing the stored mechanical energy. This phase transition‐based restoration of the original dimensions is an important contributor to the self‐healing mechanism. It is important to differentiate this from the energy storage characteristic of conventional rubbers or elastomers. The molecular transformations in rubbers or elastomers occur due to fluctuations in entropy, whereas mechano‐responsive self‐healing materials undergo phase transitions due to the intake and discharge of enthalpic energy. Nevertheless, there is a notable scarcity of research on the application of these mechano‐responsive self‐healing materials in contexts where rubber is typically used to restore original dimensions. These mechano‐responsive self‐healing materials possess advantages akin to those of rubber. As a mechanical energy storage material of rubber relies on entropy, its performance significantly varies with temperature. However, the shape‐memory effect, based on enthalpy, is intrinsic to mechano‐responsive self‐healing materials, allowing them to maintain their energy storage performance more consistently across high and low temperatures.^[^ ^60^ ^]^

Third, because self‐healing is typically associated with living organisms rather than inanimate objects, these materials have the potential to revolutionize the fields of medical implants and tissue engineering by playing a complementary role in regenerating physical damage, ultimately enhancing the performance and durability of these applications.

**Figure 3:**
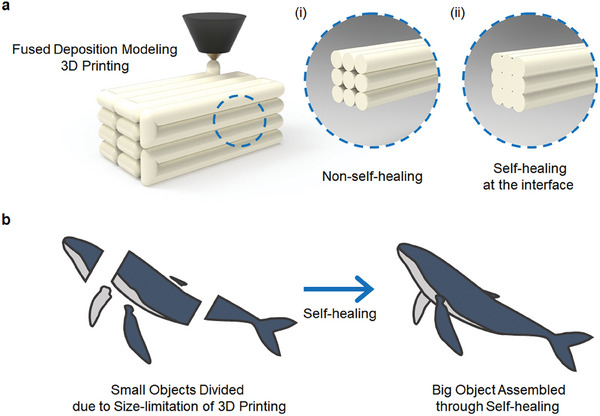
a) Schematic image of fused deposition modeling 3D printing: i) divided interface of non‐self–healing material filaments, and ii) interfused interface of self‐healing material. b) Advantages of 3D printers using self‐healing materials: overcoming size limitations of 3D printers.

Finally, self‐healing materials can considerably affect product manufacturing processes, particularly in the 3D‐printing domain.^[^ ^62^, ^63^ ^]^ By incorporating self‐healing materials, the need for adhesives is eliminated, leading to smoother and continuous production with fewer interfaces between the printed layers. Consequently, they enhance the structural integrity and performance of the final product (**Figure** **3**). For example, fused deposition modeling (FDM) is a 3D printing technique within the field of material extrusion. FDM builds components layer by layer, strategically depositing melted material, such as thermoplastic polymers, following a pre‐established path. A major challenge of FDM is the presence of an interface between the newly deposited layer and the pre‐existing layer. This interface can lead to mechanical failure and also results in grooves, impairing the smoothness of the object's contour. These issues could potentially be resolved entirely through the application of self‐healing plastics. Moreover, most 3D printing technologies, including FDM, have a size constraint for the objects they can produce, determined by the dimensions of the printing device. Utilizing self‐healing materials could circumvent this limitation. An object could be divided into multiple parts, printed separately, and then seamlessly connected at the interface, resulting in a single, larger object.

## 3 Food Monitoring

Recently, functional food packaging and related technologies have attracted much attention because of concerns about pandemics such as COVID‐19, driving life toward a contactless society mainly led by commodity plastics. Functional coating layers can improve the performances of pristine plastics, such as gas barriers for maintaining freshness, self‐cleaning for recycling efficiency, and antifogging for clear visibility.^[^ ^64^, ^65^, ^66^ ^]^

The principle of anti‐fogging materials is to regulate the interaction between water droplets and a solid surface via the chemical composition and physical roughness of the contacting surface to ensure appropriate wettability.^[^ ^67^, ^68^ ^]^ These antifogging coatings may lose their transparency when scratches are generated during handling, which also reduce the antifogging performance by changing the surface roughness.

**Figure 4:**
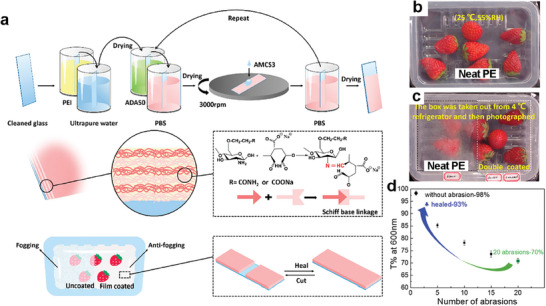
Self‐healing and antifogging properties for food packaging. a) Schematic representation of the preparation procedure for an LBL‐assembled polysaccharide coating film composed of AMCS and ADA on PEI‐treated glass. Schiff base linkage formed between amino groups of AMCS and aldehyde groups of ADA. b) Photograph of the packaging box sealed with neat PE film containing strawberries at 25 °C and 55% relative humidity. c) Photograph of the box sealed with neat PE film (left) and double‐coated PE film by polysaccharides (right) after taking it out from the refrigerator at 4 °C. d) Transmittance recovery of the coated film after various abrasions and scratches. Reproduced with permission.^[^ ^70^ ^]^ Copyright 2018, ACS.

In 2018, the Ding group developed transparent, antifogging, and self‐healing polysaccharide coating films composed of alginate aldehyde (ADA) and acrylamide‐modified chitosan (AMCS), using a layer‐by‐layer (LBL) assembly method,^[^ ^69^ ^]^ for food packaging application (**Figure** **4**a).^[^ ^70^ ^]^ This application relies on the observation that the ability of a material to self‐heal depends on the rate of the healing process and the presence of moisture, which can induce phase changes in the material. Specifically, the self‐healing function of the material is influenced by the rate of healing and the extent of moisture present. These factors can impact the overall effectiveness of the self‐healing mechanism, and thus, must be carefully considered when designing and implementing this application. By considering these critical parameters, the self‐healing capabilities of a material can be leveraged to achieve enhanced performance and durability in a wide range of applications.

The Schiff base linkage formed between the amino groups of AMCS and the aldehyde groups of ADA was used to construct films with intrinsic self‐healing properties by reversible dynamic covalent bonding. Dip coating of ADA with an oxidation degree of 50% and spin coating of AMCS with a degree of substitution of 0.54 were repeated on cleaned glass predeposited with polyethylene imine (PEI). Owing to the hydrophilic features and water‐absorbing capacities of the two polysaccharides, the coated polyethylene (PE) film exhibited antifogging properties depending on the coating thickness. Fogged packaging causes consumer inconvenience when viewing food products. Strawberries in the box sealed with the neat PE film could be clearly seen at room temperature (Figure 4b); however, heavy fog formed on the untreated PE film when it was moved from a refrigerator (4 °C) to a warmer environment (25 °C). In contrast, no fog was formed on the double‐coated (inner and outer) PE film, which retained its original transparency (Figure 4c). Interestingly, reconstruction of the reversible Schiff base linkage within the films resulted in excellent and repetitive scratch‐healing ability with the help of a water stimulus.^[^ ^71^, ^72^ ^]^ After 20 times of abrasion tests, the film became translucent, and the transmittance decreased from 98% to 72% at a wavelength of 600 nm because the generated scratches scattered visible light. The opaque film soon recovered its transparency to 93% (Figure 4d). The combination of excellent antifogging and self‐healing properties, driven by water‐induced swelling and reversible Schiff base linkage suggests food packaging material potential.

Delivering fresh food under a secure cold supply chain is of great importance in line with the surge in the food packaging industry.^[^ ^73^ ^]^ For example, recent accidents of hemolytic uremic syndrome (commonly known as hamburger disease) and ineffective thawed messenger ribonucleic acid vaccines (Pfizer‐BioNTech and Moderna) have been caused by cold‐storage malfunctions.^[^ ^74^, ^75^ ^]^ Current food‐supply chain monitoring systems operated by diffusion‐based time‐temperature indicators (TTIs) are limited by cumbersome modularization, low temperature sensitivity, and high cost.^[^ ^76^, ^77^ ^]^

In 2020, the Oh group developed the first self‐healing–mediated TTI with highly sensitive, easy‐to‐interpret, tamper‐proof, and self‐responsive changes in transparency via the flow of thermodynamic free energy of a nanofiber mat.^[^ ^78^ ^]^ This application operates based on the principle of self‐healing, which is characterized by a reduction in the interfacial area. The sensor utilized in this application operates based on the principle that the rate of reduction of the interfacial area is dependent on the temperature, as described by the Arrhenius equation.

The sensor is designed to detect changes in temperature by monitoring the rate of interfacial area reduction, which provides valuable information on the state of the system being monitored. By leveraging the self‐healing property and temperature‐dependent behavior of the interfacial area, this application can provide accurate and reliable measurements of key system parameters.

**Figure 5:**
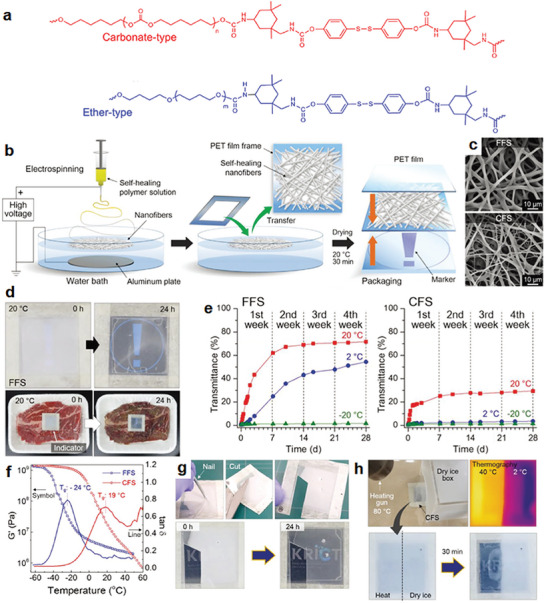
Self‐healing–mediated operation of a TTI composed of nanofiber mat. a) Chemical structures of TPUs used. Reproduced with permission.^[^ ^60^ ^]^ Copyright 2021, Springer Nature. b) Schematic representation of an electrospinning setup and assembly process. c) SEM images of the nanofiber mat for the FFS and CFS monitoring temperature changes at −20 and 2 °C, respectively. d) Visual differences in the appearance of TTI against the deterioration of chilled beef exposed at 20 °C after 24 h. e) Transmittances (wavelength of 560 nm) of FFS and CFS at various exposure temperatures for 4 weeks. f) Loss factor (tan *δ*, solid lines) and storage modulus (*G*′, symbols) curves at 1 Hz. g,h) Self‐responsive, damage‐independent (g: puncturing and cutting), and sensing partial heat (h: next to dry ice) performances of TTI. Reproduced with permission.^[^ ^78^ ^]^ Copyright 2020, Wiley.

Electrospinning of self‐healing thermoplastic polyurethane (TPU) was carried out to prepare a nonwoven nanofiber mat coagulated on a water bath to remove residual solvents, followed by fabrication in the form of a sticker veiling a “warning sign” (**Figure** **5**a,b). Original TPU materials exhibiting remarkable toughness and efficient self‐healing performances at room temperature were employed. The metathesis of aromatic disulfides afforded the contradictory properties of dynamic covalent bonds and disordered hydrogen bonding of urethane groups as supramolecular interactions.^[^ ^60^, ^79^ ^]^ Two TPUs with different chemical structures in the macrodiols were applied: ether‐ and carbonate‐type soft segments for a frozen food sensor (FFS) and chilled food sensor (CFS), respectively. Nanofiber mats for both the FFS and CFS were successfully prepared with fiber diameters of 960–1920 nm (Figure 5c). Notably, the previously reported electrospinning of self‐healing polymers was only attempted with additional components because the unsatisfactory mechanical performances of pristine self‐healing polymers resulted in an uncontrollable fusion of electrospun fibers.^[^ ^80^, ^81^ ^]^ The actual application of the manufactured TTI as a food sensor was demonstrated. The nanofiber web in the sticker was opaque at the first exposure at a room temperature of 20 °C. It revealed the hidden “warning sign” to the back of the sensor by becoming transparent by the action of self‐healing–mediated fusion of the fibers (Figure 5d). At the same time scale of 24 h, chilled beef also seemed to deteriorate due to color change. FFS and CFS monitored temperature changes from −20 and 2 °C, respectively, because the glass transition temperatures (*T*_g_s) estimated from the tan *δ* curves of FFS and CFS were −24 and 19 °C, respectively. The transmittance of FFS remained unchanged at −20 °C but steadily increased to 54% at 2 °C after 28 days. In contrast, the transmittance of CFS changed slightly at both −20 and 2 °C but changed at 20 °C (Figure 5e,f). The change in the appearance of the nanofiber mat from opaque to transparent, driven by self‐healing, can be understood in terms of thermodynamics. A fibrous structure with a larger specific surface area and more voids than the bulk film resulted in greater light scattering. Owing to the larger surface area, the higher thermodynamic free energy tended to be reduced by the self‐healing‐mediated fusion of the adjacent fibers, resulting in a transparent non‐porous film. This self‐responsive TTI functioned even when punctured and cut, and only recorded the heat history of the exposed part (Figure 5g,h). Additional advantages, including irreversibility, high flexibility, and self‐healing TTI shock resistance, eliminate the need for modularization. This type of self‐healing–mediated TTI offers a highly sensitive and easy‐to‐interpret solution for monitoring temperature changes in food packaging, which is useful for next‐generation cold chain monitoring systems and crucial for ensuring food safety and quality.

## 4 Damage‐Reporting

A sensor is a device utilized in converting specific kinds of energy into observable signals. It often pertains to a device that transduces detected signals into electrical outputs, undergoes signal processing, and subsequently facilitates visualization. However, sensors can also execute a simpler functionality by merely detecting and indicating changes. The TTI introduced previously can be regarded as a sensor from this perspective. In this section, a sensor capable of assessing the occurrence of physical damage to materials is introduced.

**Figure 6:**
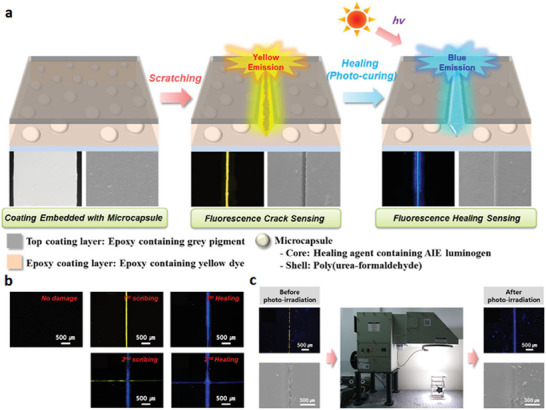
Detection system based on different fluorescent colors. a) Schematic illustration of cracking/self‐healing detectable coating system. b) Fluorescence photographs showing multi‐crack sensing and self‐healing detection. c) Real environment simulation light irradiation system under one‐sun conditions (AM 1.5G): optical microscope/scanning electron microscope images after cracking (left) and self‐healing (right). Reproduced with permission.^[^ ^82^ ^]^ Copyright 2018, Elsevier.

The Park group (2018) reported a damage‐reporting coating material using two different fluoresced colors (**Figure** **6**).^[^ ^82^ ^]^ The coating system was composed of a grey‐colored epoxy layer (top coating), yellow‐fluorescent‐dye–containing epoxy layer (crack sensing), and healing‐agent–containing microcapsule with a blue fluorescent dye based on an aggregated‐induced emission (AIE) effect (recovery‐sensing) (Figure 6a).

The authors applied an epoxy coating containing yellow fluorescent dye underneath the topcoat. As a result, a color‐detecting system capable of detecting damage history was constructed, where the underlying coating becomes visible upon damage to the coating material. Additionally, microcapsules embedded within the coating containing blue fluorescent AIE luminogen enabled visualization of the healed regions following photo‐curing, exhibiting self‐healing behavior. Furthermore, it was confirmed that such changes occurred independently depending on the moment of damage occurrence (Figure 6b).

State detection based on color changes relies on the fact that the epoxy resin containing fluorescent dye exhibits a yellow color under UV light, while the healing agent demonstrates a significant increase in fluorescence intensity upon curing under 365 nm UV light. These transformations were observed not only under specific wavelengths of UV light but also under real environmental conditions, such as one sun condition (AM 1.5G) as shown in Figure 6c.

When this system is applied, it is possible to check at a glance whether the target is damaged and whether the coating has been restored to the area.

## 5 Radiation Shielding

The self‐healing properties of protective layers generally contribute to the mechanical recovery of fractured materials. Additional electrical healing is possible when insulating polymer matrices reconcile conductive additives such as Ag nanowires (NWs) and carbon nanotubes.^[^ ^83^ ^]^ Combined composites have mostly been introduced and applied for self‐healable electronic skin sensing external stimuli, such as physical pressure, temperature, and moisture, similar to human skin.^[^ ^84^ ^]^ In addition to the aforementioned strategy, radiation shielding applications have recently emerged.

**Figure 7:**
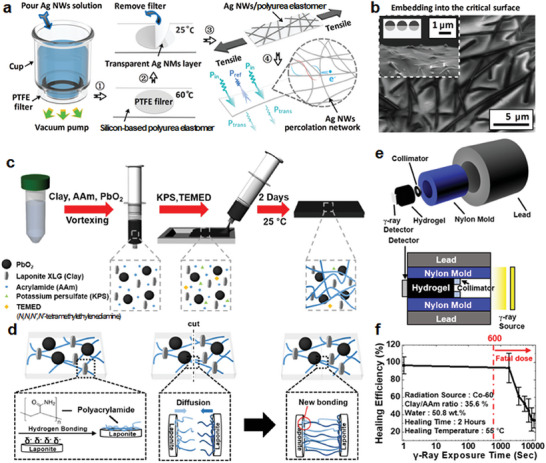
Self‐healing for radiation shielding. a) Schematic fabrication of Ag NWs embedded in a self‐healable silicon‐based polyurea elastomer by a vacuum filtration and transfer method for EMI shielding. b) SEM images show partial embedding of the Ag NWs into the polymer substrate. Reproduced with permission.^[^ ^85^ ^]^ Copyright 2021, RSC. c) Schematic preparation of a self‐healing hydrogel/metal oxide composite for γ‐ray shielding. d) Adsorption of polyacrylamides drives a self‐healing mechanism on the surface of Laponite by hydrogen bonding. New hydrogen bonding between polyacrylamide chains and Laponite by thermal diffusion progresses recovery. e) Schematic representation of the experimental measurement apparatus of γ‐ray transmission. f) Self‐healing property of hydrogel by exposure time. The red dotted line indicates the minimum detrimental γ‐ray radiation dose to humans. Reproduced with permission.^[^ ^88^ ^]^ Copyright 2020, Springer Nature.

In 2021, the Fu group utilized classical Ag NWs to prepare self‐healable, transparent, and stretchable electromagnetic interference (EMI) shielding materials.^[^ ^85^ ^]^ EMI occurs when a device is exposed to an electric or magnetic field, whereby the interference caused by this phenomenon can disrupt the normal function of the device. The vacuum filtration and transfer method elicited a robust interfacial adhesion between the Ag NWs and polymer matrix.^[^ ^86^ ^]^ EMI shielding materials are highly effective in blocking electromagnetic waves; however, their effectiveness can be significantly reduced when voids or seams occur. To ensure optimal performance, a seamless and tight coating that prevents gaps or openings from forming is essential. As previously mentioned, one of the key characteristics of self‐healing is the ability to minimize such gaps or openings, which can occur owing to various factors such as wear and tear and impact damage. By utilizing self‐healing materials, it is possible to maintain the integrity of the EMI‐shielding coating and prevent any gaps or seams from forming, thereby ensuring that the coating remains highly effective in blocking electromagnetic waves. This is particularly important in applications in which EMI shielding is critical, such as electronic devices and aerospace systems. First, Ag NWs dispersed in deionized water were filtered through polytetrafluoroethylene. A self‐healing silicon‐based polyurea elastomer film was covered and heated to 60 °C to induce semi‐embedding of the Ag NWs on the polymer surface (**Figure** **7**a,b). Even after coating with the Ag NWs, the composite exhibited high transparency (77.5%) and sufficient EMI shielding efficiency (>31 dB). Ag NWs screen out EMI because of their electrical conductivity; however, the shielding efficiency decreases when damage breaks the conductive network and increases the resistance. Therefore, recovering the connection between each Ag NW using a self‐healing polymer matrix is essential for maintaining EMI shielding efficiency.

Radiation shielding using soft materials and non‐heavy metals protects the human body from radiation exposure.^[^ ^87^ ^]^ Gamma (γ)‐ray ionizing radiation is sufficiently intense to penetrate and adversely affect the human body. In 2020, the Sun group reported an intrinsic self‐healable soft shield for γ‐rays by preparing a polyacrylamide‐based hydrogel composite containing lead dioxide nanoparticles for shielding and Laponite clay for self‐healing mediated by hydrogen bonding.^[^ ^88^ ^]^ The particles were incorporated into the stock of the acrylamide monomer and nanoclay, and polymerization was subsequently conducted using a thermal radical initiator (Figure 7c). The physical cross‐linking of polyacrylamides with clays via hydrogen bonding imparted self‐healing properties. Through cutting and rejoining, the grafted polymer chains diffused to the other side of the cut gel and formed additional hydrogen bonds (Figure 7d). The nanocomposite effectively attenuated γ‐rays. Under proper experimental apparatus for measuring the degree of shielding, the hydrogel exhibited a high attenuation coefficient of 0.1343 cm^−1^. It maintained its self‐healing property after γ‐ray exposure for up to 1800 s (Figure 7e,f). Notably, although the physical properties, including self‐healing, can be negatively affected by a large dose of radiation exposure on the hydrogel, the composite can be used to protect the human body under a mild radiation dose. A detrimental dose of 10 Gy for humans was converted to 600 s of γ‐ray exposure at a distance of 30 cm with a 1.25 meV Co‐60 source.

The use of self‐healing materials has expanded beyond mechanical recovery and can contribute to radiation shielding. Recent studies have shown the potential of combining conductive additives with insulating polymer matrices to create self‐healing EMI shielding materials. A soft shield against γ‐ray radiation was also developed using a polyacrylamide‐based hydrogel composite containing lead dioxide nanoparticles and Laponite clay for self‐healing mediated by hydrogen bonding. These self‐healing materials have the potential to offer convenient and effective protection from radiation exposure. However, it remains unclear whether the radiation shielding effect was improved by self‐healing, damage release, or stress.

## 6 Acoustic Conservation

Self‐healing technology has proven to be highly effective in maintaining the quality of acoustic devices such as optoacoustic transducers and thermoacoustic loudspeakers. For example, optoacoustic transducers in the ultrasound device family generate acoustic waves by the photoacoustic effect. Specifically, when the inorganic/polymer nanocomposites are irradiated by a pulsed laser, local heating occurs followed by periodic thermal expansion. Acoustic generators are often subjected to constant sound waves that can create microdefects and ultimately degrade their performance over time. One of the key features of self‐healing materials is their ability to continuously remove fine defects, which can help maintain the performance of the acoustic generator over a longer period. By leveraging the self‐healing capabilities of the material, it is possible to mitigate the effects of stress and wear on the acoustic generator, thereby resulting in improved reliability and longevity. This is particularly important in applications where acoustic generators are critical, such as medical ultrasound devices and industrial inspection systems, where reliable and consistent performance is essential.

**Figure 8:**
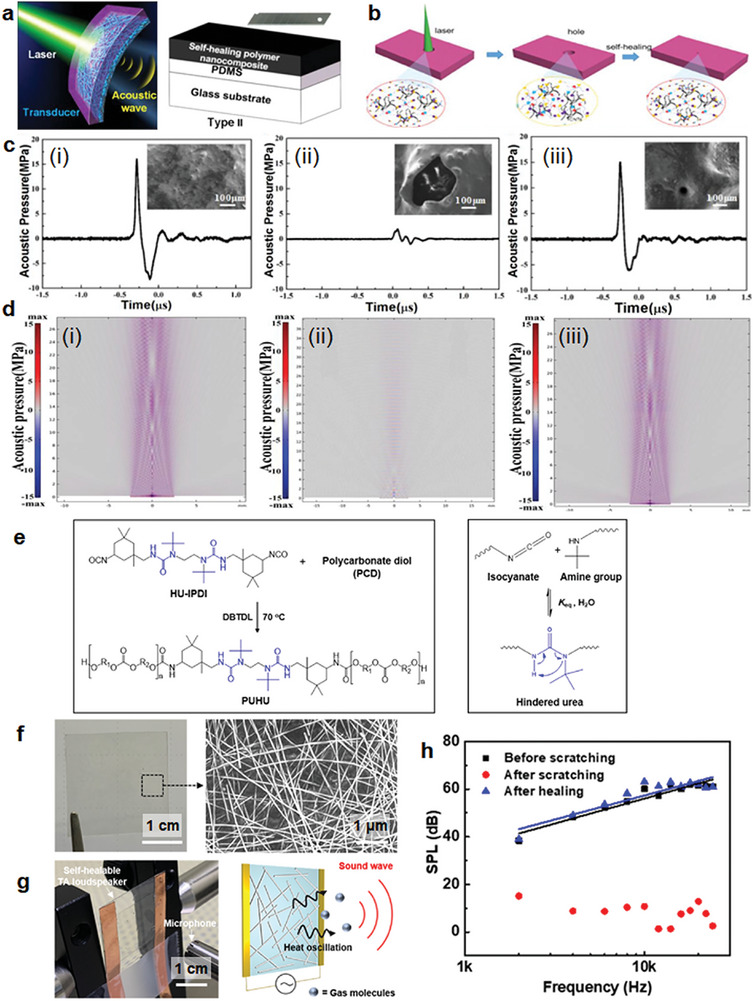
Self‐healing for acoustic conservation. a) Schematic representation of a self‐healing optoacoustic transducer and b) self‐healing process after damage by a high‐energy laser. c) Acoustic pressure, SEM images, and d) acoustic pressure simulation of the optoacoustic device at its original state (i), damaged by laser (ii), and after self‐healing (iii). Reproduced with permission.^[^ ^89^ ^]^ Copyright 2020, Elsevier. e) Synthetic scheme and self‐healing mechanism for poly(urethane‐hindered urea). f) Photograph and SEM images of a prepared electrode for a thermoacoustic loudspeaker. g) Photograph and schematic mechanism of a fabricated thermoacoustic loudspeaker. h) SPL values depend on the thermoacoustic loudspeaker sound frequency before/after scratching and healing. Reproduced with permission.^[^ ^90^ ^]^ Copyright 2020, ACS.

In 2020, the Zhu group reported a self‐healable optoacoustic transducer prepared from a three‐layered structure of a poly(urea‐urethane) elastomer cured with carbon nanotubes/polydimethylsiloxane (PDMS)/glass (**Figure** **8**a).^[^ ^89^ ^]^ The fabricated device produced high‐intensity ultrasound (29.2 MPa) with 20 mJ pulse^−1^ laser energy; the performance retained 92.3% of the original sound pressure after ten repair cycles. High‐energy laser causes physical damage, and thus, after laser irradiation (50 mJ pulse^−1^ for 5 min), a 500 µm‐sized wound appeared on the nanocomposite (Figure 8b,c). The damaged device recovered its original morphology and acoustic pressure to 94.1% of its original value after 12 h of self‐healing at room temperature. In addition, the acoustic pressure simulations are in good agreement with the experimental results, indicating the reliability of this optoacoustic device in enduring external physical or laser‐induced damage (Figure 8d).

In 2020, the Ko group fabricated thermoacoustic loudspeakers that generated sound by the temperature oscillation of the surrounding vibrating air based on Ag NW/poly(urethane‐hindered urea) conductive electrodes.^[^ ^90^ ^]^ Thin film‐type thermoacoustic loudspeakers can be used in wearable electronic devices; however, they are vulnerable to external damage, resulting in limited possibilities. Thus, first, polyurethane‐bearing bulky urea bonds were designed to induce dynamic bonding between the isocyanate and amine groups for self‐healing (Figure 8e). Ag NWs were spin‐coated onto a polymer‐coated poly(ethylene naphthalate) substrate to prepare highly transparent self‐healable electrodes (Figure 8f). The working principle began with heat oscillation by Joule heating from an applied alternating current (AC) voltage.^[^ ^91^, ^92^ ^]^ Surrounding gas molecules vibrated and made sounds at twice the frequency of the applied voltage frequency (Figure 8g). The sound pressure level (SPL) produced by the fabricated loudspeaker at a frequency range of 2–20 kHz showed excellent recovery after scratching and healing processes; SPL values of 35.5–64.2 dB decreased below 25 dB after scratching, then almost recovered to 35.0–65.6 dB after self‐healing at 95 °C and 80% relative humidity (Figure 8h). This indicates that the damage‐tolerant behavior of the self‐healable thermoacoustic loudspeaker can be applied to flexible and wearable acoustic electronics with strong durability.

## 7 Biomedical Support

**Figure 9:**
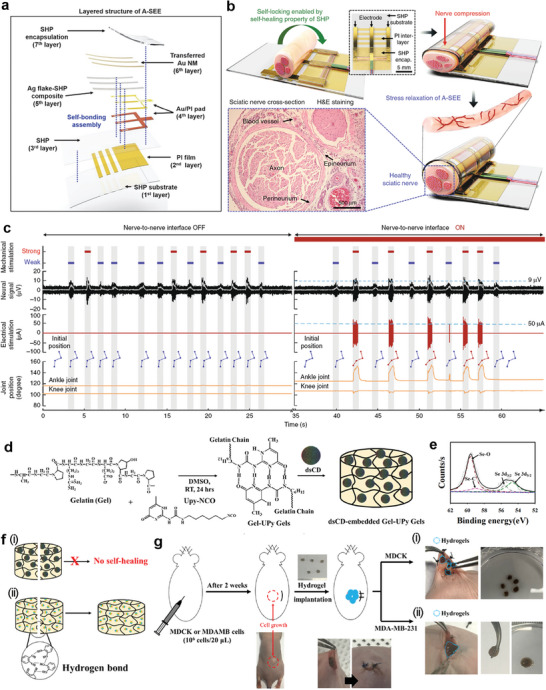
Self‐healing materials for in vivo biomedical support. a) Schematic illustration of the layered structure of the A‐SEE ensemble and b) schematic and H&E stained image of A‐SEE on the rat sciatic nerve tissue showing self‐locking and stress relaxation. c) Mechanical stimulation detected neural signals and neuromodulation induced by electrical stimulation through the nerve‐to‐nerve interface. Reproduced with permission.^[^ ^95^ ^]^ Copyright 2020, Springer Nature. d) Synthesis of Gel‐UPy/dsCD hydrogels and e) XPS analysis of cancer‐cell–embedded hydrogels. f) Self‐healing mechanism of i) unhealable Gel‐UPy/dsCD hydrogels in pH 7.4 PBS and ii) self‐healed Gel‐Upy/dsCD hydrogels via hydrogen bonding after GSH and H_2_O_2_ treatments. g) Procedure of the in vivo cancer detection test and photographic images of hydrogels after 1 h exposure to i) normal and ii) cancer tissue environments. Reproduced with permission.^[^ ^100^ ^]^ Copyright 2020, ACS.

Attempts to utilize self‐healing materials for biomedical applications are currently in progress. In the case of implantable self‐healing prostheses, they have the ability to adapt to the stresses caused by changes in the human body throughout their life cycle. This capability effectively reduces the need for surgical intervention.^[^ ^93^, ^94^ ^]^ Most studies have focused on the development of biocompatible hydrogel‐based E‐skins, sutureless dressings, and electronic medicine. The Son group (2020) developed an electronic medicine with self‐healing behavior as the principle of device assembly.^[^ ^95^ ^]^ The developed adaptive self‐healing electronic epineurium (A‐SEE), shown in **Figure** **9**a, has a layered structure that includes electrodes that can transfer electrical signals between the self‐healing polymer (SHP) layers. In addition, viability was satisfied, owing to the induced Au nanomembrane‐Ag flake‐SHP composite layer to prevent Ag leakage. The A‐SEE, which enclosed the nerve and assembled based on its self‐healing ability, interacts with the sciatic nerve, as shown in Figure 9b. It is suitable for use in the body because no other adhesives are required. Biocompatible composites can also be utilized. The A‐SEE has superior stress relaxation properties compared with PDMS, which is widely used in bioapplications.^[^ ^96^, ^97^ ^]^ This characteristic suggests that A‐SEE can be utilized as a chronic bidirectional peripheral neural interface based on the modulus‐adaptability of its physical properties.^[^ ^98^, ^99^ ^]^ The control of a nerve was successfully achieved using the A‐SEE. A nerve‐to‐nerve interface has bidirectional interactive characteristics. When an external stimulus is applied to the nerve of the foot, the intended electrical stimulus causes the opposite knee or ankle to assume a specific position. The stimulus‐reaction system through the nerve‐to‐nerve interface worked well, as shown in Figure 9c. Only neural signals were collected by stimulation, and the leg did not move when the interface was turned off. However, the electrical stimulation interface was applied to the opposite leg in connection with the collected signal, and the knee/ankle moved as designed. The stress caused by nerve movement was relaxed, and the system worked without any problems. Self‐healing is a unique characteristic of living organisms that distinguishes them from nonliving entities. However, for biological tissues to reattach after fracture, auxiliary materials are often required in addition to intrinsic biological functions. Nevertheless, self‐healing materials can attach to biological and cellular tissues in vitro and in vivo, offering significant potential for biomedical applications. By mimicking the inherent regenerative capabilities of living systems, self‐healing materials are promising for a wide range of medical applications, including tissue engineering, drug delivery, and wound healing. The ability of self‐healing materials to seamlessly integrate with biological tissues can lead to enhanced healing and tissue regeneration with the potential to revolutionize the field of regenerative medicine.

In 2020, the Park group developed an in vivo cancer sensor based on self‐healing hydrogels.^[^ ^100^ ^]^ This sensor is based on the characteristic of altering the properties of materials by changing their environment. When tumor cells are present in the body, the concentrations of glutathione (GSH) and reactive oxygen species increase fourfold and tenfold, respectively.^[^ ^101^, ^102^, ^103^, ^104^ ^]^ The fundamental material is formed by the reaction between the amine and isocyanate groups in gelatin (Gel) and ureidopyrimidinone (UPy), as shown in Figure 9d. Diselenide‐containing carbon dots (dsCDs) were embedded in the Gel‐UPy hydrogel to fabricate the final product, dsCD/Gel‐Upy. In the presence of dsCD particles, the Gel‐UPy gel lost its self‐healing property after phosphate‐buffered saline (PBS) treatment because of the disturbance by the dsCDs (Figure 9f(i)). However, higher concentrations of GSH and H_2_O_2_ in cancer cells led to the cleavage of the diselenide groups;^[^ ^105^, ^106^ ^]^ newborn Se─O bonds were also observed (Figure 9e). The cleavage of the diselenide groups provides an opportunity to form more hydrogen bonds and allows dsCD/Gel‐UPy to exhibit self‐healing behavior between the catechol groups of the dsCDs (Figure 9f(ii)). In the lap‐shear adhesion test, the adhesion force between porcine skin depended on the concentration of the tumor cells (0.11 n for control, 0.40 n for 101 cells, 0.90 n for 102 cells, 1.85 n for 104 cells, and 2.50 n for 105 cells). However, the detachment forces were similar in a typical cell environment.

For the in vivo sensor test, the sensory hydrogel fragments were implanted into mouse bodies after 2 weeks of normal and tumor cell injections (Figure 9g). The shape of the inserted hydrogel was monitored an hour later. In a typical cell environment, the hydrogels retained five separate pieces when inserted (Figure 9g(i)). However, dsCD/Gel‐UPy appeared as a single hydrogel on the cancer tissue (Figure 9g(ii)). Therefore, cancer was detected using a simple method.

**Figure 10:**
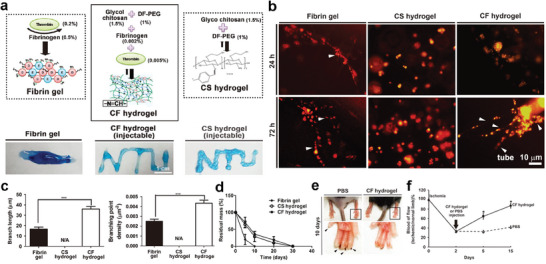
Injectable and biodegradable self‐healing hydrogel for blood capillary regeneration. a) Synthesis of the fibrin, CF hydrogel, CS hydrogel, and injectability. b) Morphology of vascular ECs encapsulated in the hydrogels after 24 and 72 h treatment. c) Branch length and branching point density of the EC tube. d) Degradation rates of the hydrogels in PBS at 37 °C. Injection of PBS and CF hydrogel into the ischemic site: e) appearance of the hindlimb after 10 days of injection and f) blood flow rates. Reproduced with permission.^[^ ^118^ ^]^ Copyright 2017, Springer Nature.

The material used must have biocompatibility and strong adhesion properties to regenerate human or animal body applications.^[^ ^107^, ^108^, ^109^, ^110^, ^111^ ^]^ In addition, a method for easy implantation is required because organisms have complex structures.^[^ ^112^, ^113^, ^114^, ^115^, ^116^ ^]^ Injectable materials, including bioinspired adhesives, may be used. However, materials are inevitably exposed to pressure from the surrounding muscles and tissues when the object moves, even after fixation. To prevent destruction from external forces, appropriate mechanical properties are necessary.^[^ ^117^ ^]^ Self‐healing is an excellent tool for supplementing this. In 2017, the Hsu group developed a blood capillary formation restorative^[^ ^118^ ^]^ and a fibrin gel of the synthesized materials, as shown in **Figure** **10**a, containing 0.2% thrombin and 0.5% fibrinogen as positive controls. The chitosan (CS) hydrogel was composed of 1.5% glycol CS and 1% telechelic difunctional poly(ethylene glycol) (DF‐PEG). The chitosan‐fibrin (CF) hydrogel was synthesized using 1.5% glycol CS, 1% DF‐PEG, 0.005% fibrinogen, and 0.002% thrombin. The CS and CF hydrogels were injected using a 26‐gauge needle. In contrast, the fibrin gel was injected using a 2 mm syringe (Figure 10a). The CF hydrogel was constructed via Schiff base crosslinking between the amine groups of CS or fibrinogen and the benzaldehyde groups of DF‐PEG.^[^ ^119^ ^]^ The CF hydrogel displayed self‐healing ability based on dynamic and reversible crosslinking.^[^ ^120^, ^121^, ^122^, ^123^, ^124^, ^125^, ^126^ ^]^ In Figure 10b,c, a blood capillary regeneration test is demonstrated. Vascular endothelial cells (ECs) penetrated the hydrogels. The ECs in the CS hydrogel aggregated after 24 h and formed spheroids after 72 h. The ECs exhibited capillary‐like morphologies in the fibrin gel after 24 h (72 h). In the CF hydrogel, spheroids were structured after 24 h, which were extended, and capillary‐like structures formed after 72 h. The vascular network in the CF hydrogel had better branch length and branching point density than did the fibrin gel. All hydrogels exhibited degradability in PBS at 37 °C PBS. The extended period of complete decomposition of the CF hydrogel was expected to support the formation of blood capillaries in the body (Figure 10d). The CF hydrogel was tested using a mouse model of hindlimb ischemia. The mouse model exhibited peripheral arterial occlusive disease (Figure 10e,f). The CF hydrogel and control sample (PBS) were injected into 2‐day ischemic mice. Ten days after injection, the PBS‐injected mice lost their nails in the disease‐occurring limb, whereas the CF‐hydrogel–injected mice did not. This could be because the blood flow in the mice injected with the CF hydrogel recovered to more than 80% between days five and 15.

Several cases of body regeneration have been reported using self‐healing materials, which is related to blood capillary restoration.

**Figure 11:**
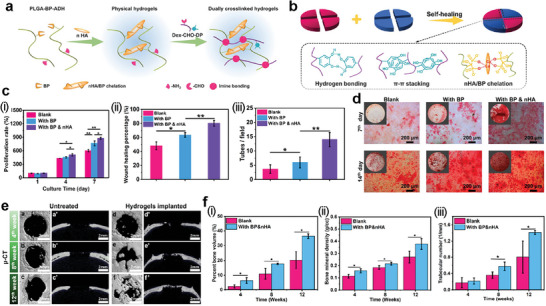
Injectable self‐healing hydrogel for bone regeneration. a) Synthesis of dual crosslinked hydrogel and b) self‐healing driving force of the hydrogel. c) Suitability of the hydrogels for bone formation assistance: i) cell proliferation rates at different times, ii) quantitative analysis of wound healing property after scratching, and iii) quantitative analysis of tube formation of ECs (blank: PLGA‐Dex hydrogels, with BP: PLGA‐BP‐Dex hydrogels, and with BP and nHA: nHA/PLGA‐BP‐Dex DC hydrogels). d) Alizarin Red S staining images for osteogenic activity evaluation of MC3T3‐E1 cells. Bone regeneration in a rat cranial defect model with or without the hydrogel treatment at the fourth, eighth, and 12th week post‐surgery: e) micro‐CT images and f) percentage bone volume (i), bone mineral density (ii), and trabecular number (iii) (blank: untreated and with BP and nHA: nHA/PLGA‐BP‐Dex DC hydrogels). Reproduced with permission.^[^ ^127^ ^]^ Copyright 2021, ACS.

In 2021, the Yin group reported a bone‐regeneration–assisted hydrogel.^[^ ^127^ ^]^ The osteogenic hydrogel was constructed using bifunctionalized precursor polymers and nano‐hydroxyapatite (nHA), as shown in **Figure** **11**a. Bisphosphonate hydrazide‐difunctionalized poly(l‐glutamic acid) (PLGA‐BP‐ADH) and aldehyde‐catechol–difunctionalized dextran (Dex‐CHO‐DP) were used as precursors. PLGA mimics the collagen in the body and functions as a matrix for bone regeneration. Bisphosphonate (BP) undergoes non‐covalent chelation with dispersed nHA. This induced physical crosslinking and prevented agglomeration of nHA. Dual‐crosslinking was performed using hydrazide (ADH) and aldehyde (CHO) via a Schiff base reaction. BP and catechol (DP) provided tissue adhesiveness, self‐healing ability, and osteoconductivity to the fabricated 3D matrix. The hydrogel was self‐healed by three driving forces: hydrogen bonding, π–π stacking, and nHA‐BP chelation, as shown in Figure 11b.

Figure 11c shows that the hydrogel is suitable for use in the body. The proliferation rate of osteoblasts (MC3T3‐E1) was also investigated (Figure 11c(i)). The rates increased rapidly to 430–535% and 623–899% on days four and seven, respectively, for the prepared hydrogels (blank: PLGA‐Dex hydrogels, with BP: PLGA‐BP‐Dex hydrogels, and with BP and nHA: nHA/PLGA‐BP‐Dex DC hydrogels). In particular, the hydrogel containing BP and nHA showed superior performance in terms of the wound healing percentage and tube formation by ECs (Figure 11c(ii),(iii)). Alizarin Red S staining images (Figure 11d) show mineralization of the extracellular matrix. The nHA/PLGsA‐BP‐Dex DC hydrogel exhibited crowded red spots on days seven and 14. An abundantly mineralized environment provides suitable conditions for bone regeneration. Micro‐CT images confirmed that hydrogel implantation was advantageous for bone formation (Figure 11e). The cranial defect in the rat was recovered by acceleration using a hydrogel assistant. The hydrogel‐implanted model exhibited outstanding values for bone volume, mineral density, and trabecular number compared with the untreated model at every observation point (Figure 11f).

## 8 Summary and Outlook: A Glimpse into the Future of Self‐Healing Materials in Industries

In summary, self‐healing materials can be broadly categorized into three branches by length scale. The trend has shifted from the “nano‐to‐micro scale,” which was easier to produce, toward the “molecular scale,” based on dynamic covalent bonds or supramolecular interaction. It is due to the development of materials that possess following attractiveness: 1) utilization in a mild condition without external stimuli, 2) utilization using common external stimuli in the use environment, and 3) secondary utilization of external stimuli. Materials with such advantages have demonstrated potential for various applications, as described in the above.

Self‐healing materials have shown immense potential across a wide range of applications, acting as a link for advancements in materials science and engineering. By exploring the practical applications of these materials, researchers can uncover new possibilities and propel industries toward a more sustainable and efficient future. Self‐healing polymers, a prominent topic in materials science and engineering, have gained considerable attention for their ability to repair damage, extend life cycles, and minimize waste and cost. Despite numerous studies exploring novel chemical designs, diverse polymer structures, and the optimization of applications in protective materials, challenges such as scalability, low robustness, and unsuitability under certain conditions have limited commercialization. To overcome such drawbacks, developments are underway in self‐healing materials possessing characteristics akin to thermoset polymers, such as CANs, which exhibit enhanced mechanical properties.

This perspective discusses the practical applications of self‐healing materials in food packaging, radiation shielding, acoustic conservation, in vivo sensing, and body regeneration, demonstrating the potential of these materials as keystones in advancing various industries. Although self‐healing polymers have different fundamental properties, they may lead to a suitable expansion of applications based on these different characteristics.

The application of self‐healing materials in functional food packaging has shown promise for improving food visibility and freshness. For instance, antifogging materials, such as transparent self‐healable polysaccharide coating films, offer excellent scratch‐healing ability and antifogging properties. Another innovative application is in cold supply chain monitoring systems, where self‐healing–mediated TTIs provide a sensitive, easy‐to‐interpret, and tamper‐proof solution to ensure food safety and quality.

Radiation shielding is another area in which self‐healing materials play a crucial role. By combining self‐healing properties with conductive additives, protective layers can recover mechanically and electrically. Studies have demonstrated the effectiveness of self‐healable, transparent, and stretchable EMI shielding materials that rely on the self‐healing polymer matrix to maintain their shielding efficiency. Additionally, self‐healable soft shields for **γ**‐radiation have been developed, demonstrating their potential for convenient and effective radiation protection.

In the field of acoustic conservation, self‐healing technology has proven valuable in maintaining the quality and durability of acoustic devices such as optoacoustic transducers and thermoacoustic loudspeakers. Studies have shown that self‐healable optoacoustic transducers can recover their original morphology and acoustic pressure after damage, whereas thermoacoustic loudspeakers with conductive electrodes demonstrate excellent SPL recovery. These findings suggest that self‐healing materials can enhance the performance of flexible and wearable acoustic electronic devices.

Finally, the biomedical field has witnessed a surge in interest in self‐healing materials for various applications, including biocompatible hydrogel‐based E‐skin, sutureless dressings, electronic medicine, and in vivo cancer detection. The adaptive self‐healing A‐SEE demonstrates stress‐relaxation properties, enabling its use as a chronic bidirectional peripheral neural interface. Furthermore, self‐healing hydrogels have been employed in in vivo cancer sensors, injectable hydrogels for blood capillary regeneration, and hydrogels for bone regeneration, highlighting their potential for developing innovative therapeutic solutions to address various medical needs.

At the end of this perspective, we elucidate the salient aspects of the industrialization of contemporary self‐healing research and provide a forward‐looking perspective on the future of self‐healing materials and their potential applications.

As the domain of self‐healing materials progresses at a remarkable pace, researchers have endeavored to overcome existing limitations and harness their potential to revolutionize myriad industries. Recent studies have delved into several key areas, including 1) novel healing mechanisms,^[^ ^128^, ^129^, ^130^, ^131^, ^132^, ^133^ ^]^ 2) enhanced healing efficiency,^[^ ^134^, ^135^, ^136^, ^137^ ^]^ and 3) scalability and affordability.^[^ ^138^, ^139^, ^140^, ^141^ ^]^ The exploration of innovative self‐healing mechanisms and their integration with established materials will facilitate the development of advanced multifunctional self‐healing systems. Merging self‐healing properties with other desirable traits, such as self‐cleaning, anti‐corrosion, and antimicrobial features, will augment the applicability of these materials across diverse industries. Current research endeavors to improve the efficiency of the healing process in terms of speed, repeatability, and completeness of repair. Fabricating materials capable of recovering from damage more rapidly and multiple times with minimal loss of functionality will significantly enhance their commercial potential. One of the primary challenges in adopting self‐healing materials is the ability to scale up production while maintaining affordability. Research efforts should concentrate on devising cost‐effective and scalable manufacturing processes to satisfy the growing demand for self‐healing materials across various sectors. The three major studies currently underway will facilitate the seamless integration of self‐healing materials into industries where they have not yet been commercialized.

**Figure 12:**
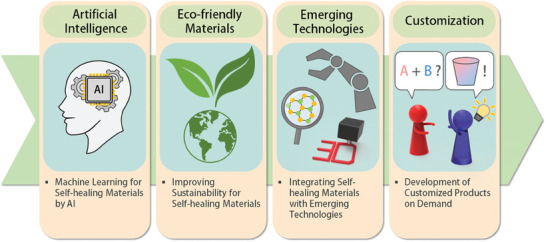
Developing process for self‐healing materials on demand by computational techniques.

In the future, self‐healing materials are anticipated to feature prominently in research that incorporates cutting‐edge technology and addresses pressing societal challenges. Some of the most promising research directions include enhancing environmental sustainability, leveraging advanced computational techniques, integrating with emerging technologies, and tailoring materials for specific applications (**Figure** **12**). These captivating developments and their potential future impact are discussed below.

Self‐healing materials are currently being developed for a greener future. With climate change and environmental degradation becoming increasingly important issues, the demand for sustainable materials has become paramount. Researchers are now prioritizing the development of ecofriendly, biodegradable, and self‐healing materials derived from renewable resources.^[^ ^142^, ^143^, ^144^, ^145^, ^146^, ^147^ ^]^ By mitigating the environmental impact of these materials, scientists aim to foster a greener future for industries ranging from automotive to construction. For instance, creating self‐healing materials from plant‐based polymers, such as cellulose or chitosan, will not only provide a renewable source for these materials but also help reduce reliance on their non‐renewable petroleum‐based counterparts. These advances in sustainable self‐healing materials will contribute to a circular economy in which waste is minimized and resources are efficiently utilized.

In addition, the greatest advantages of self‐healing materials, repairability, and longer lifetime, could fundamentally reduce material consumption. (Re)producing raw materials and processing them to utilize them from nature or while recycling them requires high costs and energy inputs. Given that these resources can be saved, the industrialization of self‐healing materials can contribute to a greener future.

Unfortunately, there are a limited number of studies available that have investigated indicators suggesting the industrialization potential of self‐healing polymers. Only a few studies have conducted a representative analysis known as life cycle assessment (LCA). In one study, a comparison was made between non‐self–healing poly(lactic acid) and self‐healing PU used in FDM‐type 3D printing, analyzing stages such as raw material manufacturing, filament manufacturing, 3D printing, transportation, and waste.^[^ ^148^ ^]^ The results confirmed that self‐healing materials exhibit higher levels of circularity and safety compared to non‐self–healing materials. Specifically, self‐healing materials demonstrated a 43.52% level in global warming potential (GWP; kg CO_2_ eq), 52.65% level in marine ecotoxicity (ME; kg 1,4‐dichlorobenzene), 37.41% level in stratospheric ozone depletion (SOD; kg CFCl_3_ eq), and 30.71% level in water consumption (WC; m^3^) compared to their non‐self–healing counterparts.

Additionally, LCA was conducted on the material of propeller blades used in unmanned aerial vehicles.^[^ ^149^ ^]^ A glass‐fiber–reinforced polyamide with self‐healing material coating was compared to a non‐coated sample as a control. Injection molding, coating, use, transportation, and waste stages were investigated. Similar to the previous case, GWP, ME, SOD, and WC were calculated. The self‐healing material coated propeller blades showed a 56.31% level in GWP, 94.80% level in ME, 37.86% level in SOD, and 29.32% level in WC compared to the non‐self–healing blades.

In both cases, GWP is particularly noteworthy. A reduction of the carbon footprint is an important issue in modern society. The results demonstrate that the use of self‐healing materials can be presented as an alternative for reducing CO_2_ emissions. Moreover, increased repetitive use can disrupt the periodicity of the “material production—product manufacturing—disposal—repurchase (reproduction)” cycle. By extending the period of repeated material circulation, the amount of CO_2_ generated during the process could be drastically reduced. The use of self‐healing materials also holds high economic value as it can help reduce carbon taxes associated with maintaining existing systems. These findings reveal that the utilization of self‐healing polymers is valuable not only in addressing greenhouse gas‐related issues but also in addressing environmental hazards, water consumption cycles, and ozone depletion.

Moreover, these materials not only contribute to the reduction of environmental degradation but also offer potential cost‐saving benefits for consumers and incentives for producers in terms of reducing carbon taxes through decreased greenhouse gas emissions. Moreover, as the commercialization of self‐healing materials continues to gain momentum, the scope of LCA research pertaining to these materials is anticipated to expand. Consequently, such research endeavors will further illuminate the value and potential of self‐healing materials across various industries.

Advanced computational techniques, such as machine learning and molecular dynamics simulations, are poised to become game changers for enhancing the performance of materials.^[^ ^150^, ^151^, ^152^, ^153^ ^]^ These cutting‐edge computational methods have the potential to revolutionize the design and optimization of materials. Therefore, it is easy to predict that the advanced computational techniques widely used in materials science will also be applied to self‐healing materials by providing a deeper understanding of their underlying healing mechanisms, ultimately enabling the engineering of materials with improved properties and performances.

Specifically, candidate monomers with the self‐healing properties are numerous, and synthesized polymers may have a wide range of properties. There may be flexible, stretchable elastomers with poor toughness but very fast self‐healing rates, while there may also be materials with superior mechanical properties but relatively slow self‐healing rates. These characteristics are trade‐offs, and materials with intermediate properties also exist. Among them, there is no absolutely perfect material that can be applied in all fields. There is a suitable range of properties for each use. From a materials engineering perspective, it is important to utilize the unique properties of materials in the right place. Advances in computational technology will make this easier and are expected to play an important role in increasing the utilization of self‐healing materials.

Artificial intelligence (AI) and molecular dynamics simulations can identify patterns and correlations in vast datasets, thereby enabling scientists to predict and optimize the behavior of self‐healing materials. This approach accelerates the development process, resulting in more effective and efficient materials tailored to the specific needs of diverse materials applications. As a partial example that fits this perspective, the Liu group conducted a molecular dynamics simulation study of self‐healing vitrimers.^[^ ^154^ ^]^ The authors investigated the effects of the bond swap energy barrier and temperature on various characteristics of vitrimers, such as bond exchange reactions, uniaxial stress–strain behavior, stress relaxation, self‐healing performance, and extrusion reprocessing.

As the next frontier, the convergence of self‐healing materials with emerging technologies, such as nanotechnology, robotics, and 3D printing, will engender a new wave of innovative solutions.^[^ ^10^, ^62^, ^155^, ^156^, ^157^, ^158^, ^159^ ^]^ Embedding self‐healing capabilities into soft robotics or 3D‐printed structures allows researchers to develop resilient and adaptive systems that mimic biological processes. As mentioned earlier with the examples of TTI or damage‐reporting sensor,^[^ ^78^, ^82^, ^160^, ^161^ ^]^ integrating self‐healing materials into nanotechnology‐enabled devices could lead to the development of smart sensors capable of detecting and repairing damage in real time.

Similarly, the incorporation of self‐healing properties into soft robotic systems can result in more robust and adaptable machines capable of withstanding harsh environments, thereby improving efficiency and reducing maintenance costs. As previously detailed (Figure 3), within the field of soft robotic fabrication, individual components of the final product can be independently produced using methodical 3D printing strategies. This approach facilitates the enhancement of the final product's size during the post‐assembly phase of the separately fabricated parts, thereby eliminating the requirement for interfacial adhesives during soft robot assembly.

The key to the success of self‐healing materials lies in their tailoring for specific applications. As self‐healing materials continue to evolve, application‐specific research will become a critical area of focus. By optimizing these materials for targeted applications, such as aerospace, electronics, medical devices, and infrastructure, researchers can ensure that the unique requirements of each application are met. For instance, self‐healing materials tailored for aerospace applications can reduce maintenance costs and increase the longevity of aircraft components. In the medical field, optimized self‐healing materials can lead to innovative therapeutic solutions such as biocompatible hydrogels for tissue regeneration and sutureless dressings for wound healing.

The future of self‐healing materials is bright, with promising research directions addressing sustainability, computational techniques, integration with emerging technologies, and application‐specific optimization. These advances will undoubtedly revolutionize industries and significantly improve the quality of life, paving the way for a more resilient and sustainable world.

## Conflict of Interest

The authors declare no conflict of interest.
